# *Lysimachia yulongensis* (Primulaceae), a new species in *Lysimachia* sect. *Pumilae* from Yunnan, China

**DOI:** 10.3897/phytokeys.270.177773

**Published:** 2026-02-02

**Authors:** Ming-yun Sheng, Wu-hai Yang, Tian Shuai, Yu-fan Chen, Li-juan Yin, Zhi-kun Wu

**Affiliations:** 1 Department of Pharmacy, Guizhou University of Traditional Chinese Medicine, Guiyang, 550025, Guizhou, China Department of Pharmacy, Guizhou University of Traditional Chinese Medicine Guiyang China; 2 Tianzhu County People’s Hospital, Tianzhu County, 556600, Guizhou, China Tianzhu County People’s Hospital Tianzhu County China; 3 Lijiang Vocational and Technical College, Lijiang, 674100, Yunnan, China Lijiang Vocational and Technical College Lijiang China

**Keywords:** Conservation status, diversity, nomenclature, taxonomy, yu long zhen zhu cai, Yunnan

## Abstract

*Lysimachia yulongensis* Z.K.Wu & Ming Y.Sheng, a new species of Primulaceae from Yunnan, China, is described and illustrated. Morphological evidence places *L. yulongensis* within *Lysimachia* sect. *Pumilae*, which is distinguished by dwarf plants with prostrate or nearly erect habits, few flowers clustered at the stem apex or solitary in the leaf axils near the apex, lanceolate sepals, elliptical and dorsifixed anthers and styles approximately as long as the stamens. The new species is characterised by a strongly stoloniferous growth habit, with creeping stems serving a vegetative function as stolon and terminating in leaf rosettes during fruiting; leaves that are narrowly elliptic to oblanceolate or spatulate; and flowers solitary in the leaf axils along obliquely ascending stems. Information on the distribution, phenology and conservation status of the new species is also provided.

## Introduction

*Lysimachia* L., a genus in the tribe Lysimachieae Benth. & Hook. f., is one of the largest and most widely distributed genera in the family Primulaceae (Chen and Hu 1989; Fang 2003). Subsequent molecular phylogenetic and morphological studies led to the transfer of *Lysimachia* and other genera of Lysimachieae to the family Myrsinaceae (Anderberg and Ståhl 1995; Källersjö et al. 2000; Mast et al. 2001; Anderberg et al. 2002, 2007), prior to the eventual incorporation of Myrsinaceae into Primulaceae (APG IV 2016). *Lysimachia* comprises approximately 288 species distributed worldwide (POWO 2025). Most species occur in temperate and subtropical regions of the Northern Hemisphere, with a limited number found in Africa, Latin America and South America (Hu and Kelso 1996; Liu et al. 2014a). China harbours particularly high species diversity within the genus *Lysimachia*. According to the Flora of China, approximately 138 species were recorded (Hu and Kelso 1996). With intensified botanical exploration in the country, 18 additional species have been discovered and described over the past two decades (Shao et al. 2004, 2006; Liu et al. 2014b, 2020; Zhou et al. 2015; Wang et al. 2016, 2018; Yan et al. 2017, 2023; Huang et al. 2019; Mou et al. 2020; Tang 2020; Yi 2020; Ju et al. 2021; Ke et al. 2021; Lu et al. 2021; Bo et al. 2022; Kottaimuthu 2023). Consequently, China is now recognised as home to approximately 156 species of *Lysimachia*.

During botanical expeditions to Yulong Snow Mountain in Yunnan in July and September 2016, we encountered a unique population of *Lysimachia*. At the flowering stage, the plants exhibited prostrate stems; by the fruiting stage, however, they developed a distinctly stoloniferous habit, with stem tips terminating in a leaf rosette and producing roots, indicating a vegetative function. The flowers were solitary in the leaf axils along the stems. In subsequent years, we revisited the site during both flowering and fruiting phases to better characterise the taxon. We also examined *Lysimachia* specimens collected from Yulong Snow Mountain deposited in the Herbaria KUN and PE and found that this plant had previously been identified as *Lysimachia parvifolia* Hemsl. *L. parvifolia* is characterised by erect or diffuse stems that arise from the base of a leaf rosette and racemose inflorescences. In contrast, the newly-discovered plants have creeping stems that root at the nodes near the apex, clearly serve a vegetative function as a stolon and terminate in a leaf rosette during fruiting. Moreover, all flowers are solitary in leaf axils and borne obliquely upwards along the stem, clearly distinguishing them from the racemose inflorescences of *L. parvifolia*. Through literature review and detailed morphological comparisons with related species, we confirmed that this population represents a previously undescribed species of *Lysimachia*. We, therefore, formally describe and illustrate it here as a new species.

## Materials and methods

Type specimens and living materials of the new species were collected from Yulong Snow Mountain in Yunnan, China. Morphological observations, measurements and descriptions were conducted on randomly selected living individuals. Comparative morphological analyses with closely-related species were performed using living plants of *Lysimachia prolifera* Klatt collected from the Salwin-Kiukiang Divide, as well as materials from the type localities of *Lysimachia pumila* (Baudo) Franch., together with herbarium specimens obtained from major Chinese herbaria (KUN, PE, IBSC) and digital type images accessed from E and NY (isotype of *L. prolifera* (J. D. Hooker 8, E, specimen number E00062011); holotype of *L. pumila* (P. J. M. Delavay 1091, NY, specimen number 00329494)). Relevant literature (Chen and Hu 1989; Hu and Kelso 1996) and the original protologues of *L. prolifera* and *L. pumila* were also consulted (Franchet 1895; Klatt 1866). Morphological characters of *L. yulongensis*, *L. prolifera* and *L. pumila* were measured using a Vernier caliper. The conservation status of the new species was assessed following the IUCN Categories and Criteria (IUCN Standards and Petitions Committee 2024).

## Taxonomic treatment

### Lysimachia yulongensis

Taxon classificationPlantaeEricalesPrimulaceae

Z.K.Wu & Ming Y.Sheng sp. nov.

384BC4C0-B6CD-5864-9D77-CCA1CF6D1A08

urn:lsid:ipni.org:names:77376163-1

Figs 1, 2, 3

#### Diagnosis.

The new species exhibits a floral morphology similar to that of *Lysimachia prolifera* and *L. pumila*, characterised by dwarf plants with creeping or nearly erect habits, few flowers clustered at the stem apex or solitary in leaf axils, lanceolate sepals, elliptical and dorsifixed anthers. However, it can be distinguished from these two species by several morphological features: a strongly stoloniferous growth habit, with creeping stems serving a vegetative function as stolon and terminating in a leaf rosette during fruiting; narrowly elliptic to oblanceolate or spatulate leaves; and flowers solitary in leaf axils along obliquely ascending stems.

**Figure 1.**
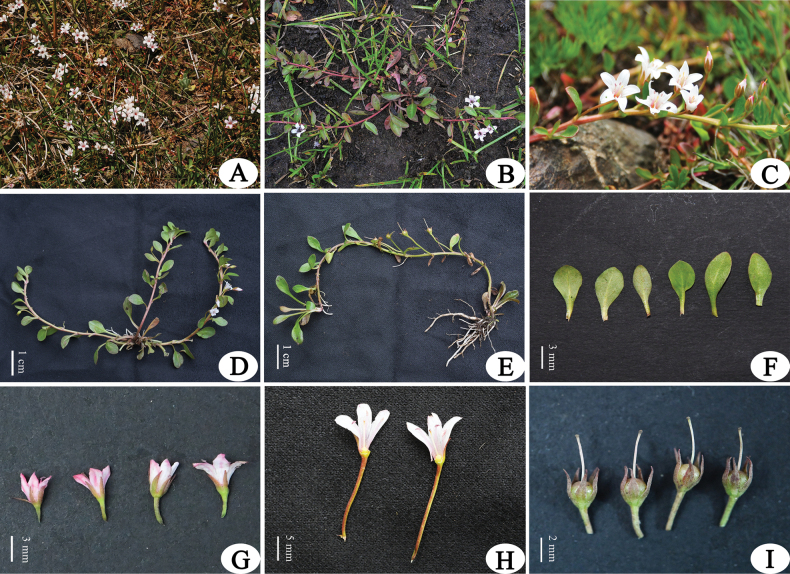
*Lysimachia yulongensis* sp. nov. **A, B**. Habitat; **C, D**. Habit during flowering; **E**. Habit during fruiting with stolons; **F**. Leaves, the left three: lower surface, the right three: upper surface; **G**. Flower with calyx; **H**. Dissected corolla showing anthers and stigmas; **I**. Young fruits. Photographed by Zhikun Wu.

**Figure 2.**
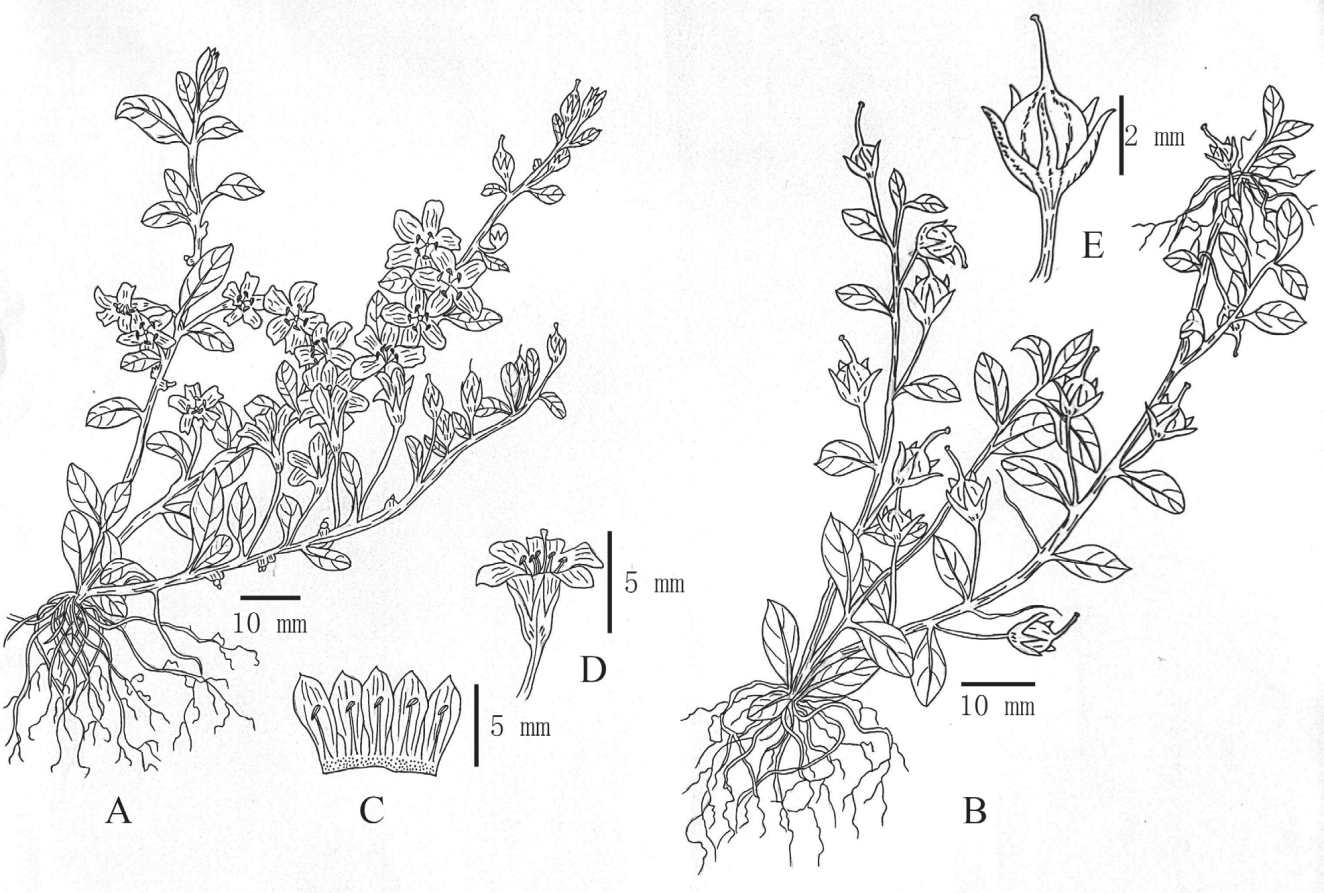
*Lysimachia yulongensis* sp. nov. **A**. Flowering habit; **B**. Fruiting habit with stolons; **C**. Dissected corolla; **D**. Flower; **E**. Fruit. Drawn by Ms. Xiangli Wu.

**Figure 3.**
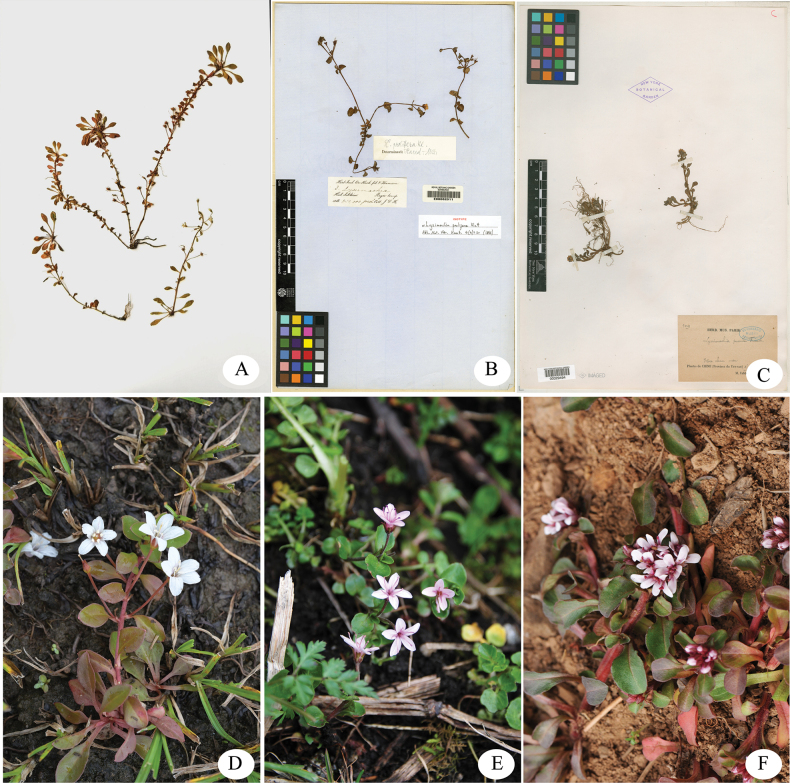
*Lysimachia yulongensis* and two morphologically similar taxa. **A**. Holotype of *L. yulongensis* (*ZKWU 2024146*); **B**. Isotype of *L. prolifera* (J. D. Hooker 8, E, specimen number E00062011); **C**. Holotype of *L. pumila* (P. J. M. Delavay 1091, NY, specimen number 00329494); **D**. *L. yulongensis*; **E**. *L. prolifera*; **F**. *L. pumila*; **D–F**. Photographed by Zhikun Wu.

#### Type.

China • Yunnan: Lijiang, Yulong County, Baisha Town, Yulong Snow Mountain; 26°57'N, 102°13'E, 3100 m alt., 1 August 2024 (fr., fl.) *Zhikun Wu ZKWU 2024146* (holotype: KUN!; isotype: KUN!).

#### Description.

A robust perennial herb, dwarf, efarinose, sparsely glandular, entirely glabrous. Rhizomes short, with numerous fibrous roots. ***Stems*** creeping, 10–20 cm long, usually much-branched from base, elongated, producing adventitious roots from apical nodes during fruiting for propagation. ***Leaves*** alternate, subsucculent, narrowly elliptic to oblanceolate or spatulate, 0.6–2.1 cm long (including petiole), 0.3–0.6 cm wide, apex obtuse to rounded, base attenuate into a winged petiole; petiole 0.2–0.7 cm long, ca. 1/3 as long as leaf blade; adaxial surface puberulent, abaxial surface sparsely covered with dark purple or black glands. ***Flowers*** solitary in leaf axils, obliquely ascending at anthesis, 5–20 flowers per plant; pedicel 0.5–2.5 cm long, longer than leaves, gradually shorter towards stem apex. ***Calyx*** lobes narrowly lanceolate, ca. 3 mm long, margins hyaline, abaxially sparsely dotted with dark purple or black glands. ***Corolla*** white to pale pink, campanulate, 5–11 mm long, divided to middle; tube ca. 2–4 mm long; lobes oblong, 3–7 mm long, 0.5–1 mm wide, eglandular, apex obtuse; stamens included, slightly shorter than corolla lobes; filaments adnate to lower part of corolla lobes, free portion ca. 2 mm long; anthers elliptic, dorsifixed, ca. 1–2 mm long; ovary ovoid, glabrous; style ca. 6 mm long. ***Capsule*** subglobose, ca. 2 mm in diameter.

#### Distribution and ecology.

**Map 1.**
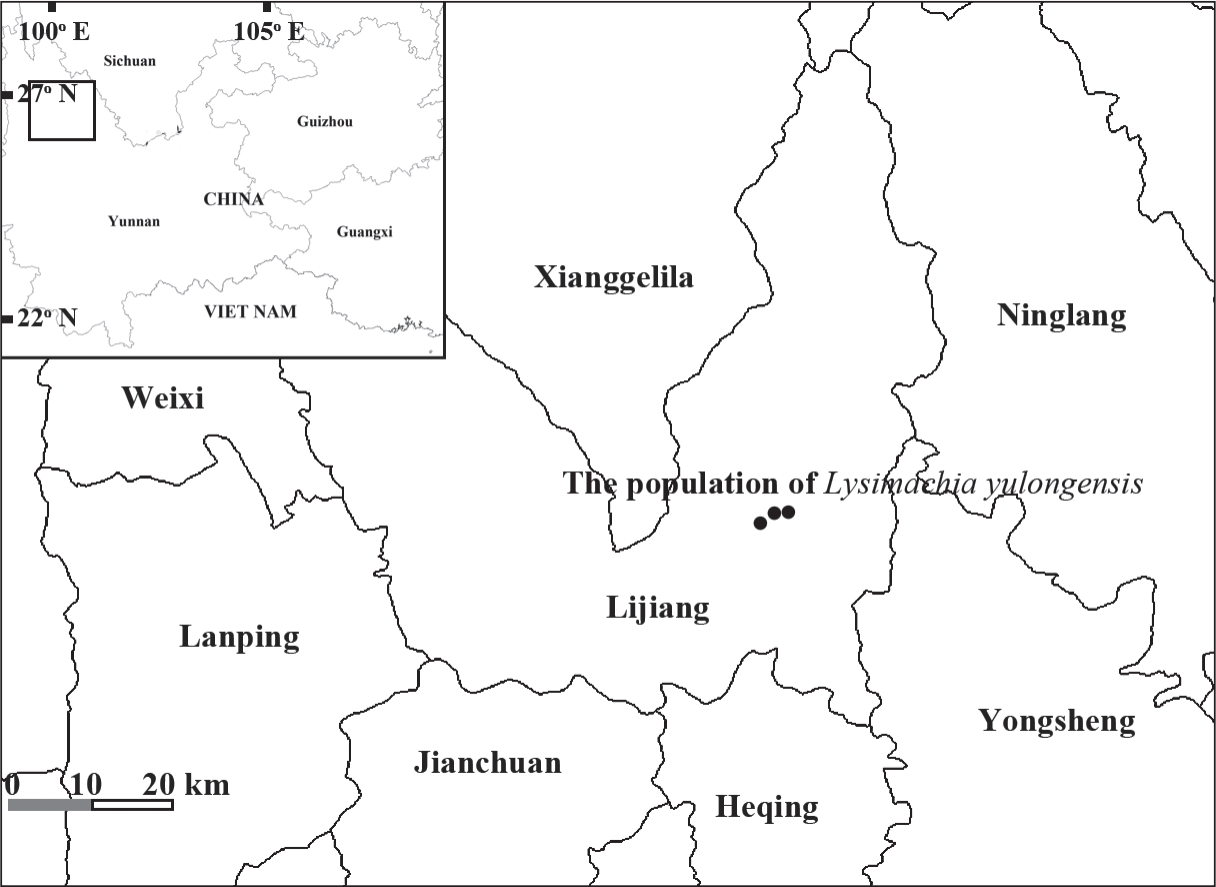
Locations of the three populations of *Lysimachia yulongensis* in Yulong Snow Mountain, Lijiang, Yunnan.

Based on specimens deposited in KUN and PE, as well as our field surveys in Yunnan and adjacent regions, *Lysimachia yulongensis* is currently known only from the vicinity of Yulong Snow Mountain in Lijiang, Yunnan, China. To date, three populations have been recorded in this area. The species occurs in wet meadows along the margins of alpine lakes and streams (Fig. 1, Map 1).

#### Phenology.

The species was observed flowering from June to August and fruiting from August to September.

#### Etymology.

The specific epithet of the new species is taken from the Chinese Pinyin ”Yulong”, the name of the mountain in north-western Yunnan, China, where the type specimen was collected (Map 1).

#### Vernacular name.

Chinese Mandarin: “yu long zhen zhu cai” (玉龙珍珠菜).

#### Provisional conservation status.

Endangered (EN B1ab(iii)). Repeated field surveys within the range of Yulong Snow Mountain and adjacent areas have revealed only three populations of *L. yulongensis*, totalling approximately 2000 mature individuals. Specimens deposited in herbaria (KUN, PE, E, NY, IBSC) further indicate that other collectors have recorded no additional populations during historical or recent fieldwork. All three known populations occur in wet meadows along alpine lakes and streams. These habitats are subject to significant anthropogenic pressure from livestock grazing and tourism development. Given these threats, the species requires urgent conservation attention and further field surveys are recommended to monitor its status.

According to our estimates, the extent of occurrence (EOO) of this species is less than 1000 km^2^. Over the past nine years, field observations have indicated a continuous decline in the quality of its habitat, primarily due to tourism activities and grazing pressure. This ongoing degradation underscores the urgent need for conservation measures to safeguard both the species and its habitat. Based on current field data and the IUCN Red List Categories and Criteria (IUCN Standards and Petitions Committee 2024), we propose that this species be classified as Endangered (EN B1ab(iii)).

#### Additional specimens examined.

China • Yunnan Province, Lijiang, Yulong Mountain, Wenhai, the type location, 8 July 1962, Zhang Aoluo and Yu Shaowen, *100524* (KUN!), 20 June 2016 (fl.), ZhiKun WU *ZKWu2016620* (KUN!), 29 August 2022 (fr.) ZhiKun WU, *ZKWu2022829* (KUN!); China • Yunnan Province, Lijiang, Yulong Naxi Autonomous County, Lashi Town, Lashihai Wetland Park [26.844759°N, 100.132004°E], 16 June 2021, Liu Ende, Zou Jiao, Wang Cunhua, Chen Zhijian, Shangguan Fazhi, *BIO10474* (KUN!); China • Yunnan Province, Lijiang, West Slope of Jade Dragon Snow Mountain [27°20'], July 1918, George Forrest *16244* (KUN!).

## Discussion

Within the diverse genus *Lysimachia*, subgenus *Palladia* (Moench) Hand.–Mazz. can be readily distinguished from other subgenera by a combination of characters: pentamerous flowers that are white, pink or rose-purple, arranged in terminal racemes (rarely solitary in the upper leaf axils or clustered at the stem apex) and filaments that are free and adnate to the upper part of the corolla tube or the base of the corolla lobes. *L. yulongensis* exhibits pentamerous, white to pale pink flowers, along with free filaments adnate to the base of the corolla lobes, confirming its placement within subgenus *Palladia*. Species with procumbent stems or stoloniferous habits are common in the genus *Lysimachia*, particularly in subgenus *Lysimachia* L. and subgenus *Idiophyton* Hand.–Mazz. However, such habits are rare in subgenus *Palladia*, where only section *Candidae* Hand.–Mazz. and section *Pumilae* (Hand.–Mazz.) Chen & C.M.Hu exhibit these growth characteristics.

In his 1928 revision of Chinese *Lysimachia* species, Handel-Mazzetti recognised ten sections within *L.* subgenus *Palladia* (Handel-Mazzetti 1928);in contrast, subsequent classifications by Chinese researchers have consistently divided the Chinese species of this subgenus into eight sections, recognising sect. *Pumilae* and sect. *Candidae* as independent sections (Chen and Hu 1979; Hao et al. 2004). Morphologically, the two sections are readily distinguishable: plants of sect. *Pumilae* are dwarf, with few flowers clustered at the stem apex or solitary in leaf axils near the apex, while the plants of sect. *Candidae* bear numerous flowers arranged in erect racemose inflorescences. Based on the creeping stems and axillary solitary flowers observed in *L. yulongensis*, this new species is assigned to *L.* sect. *Pumilae* of subgenus *Palladia*.

Sect. *Pumilae* is a small section that has been recognised across all major taxonomic systems as comprising only two species: *Lysimachia prolifera* and *L. pumila*. With the addition of *L. yulongensis*, this section now encompasses three species. However, *L. yulongensis* can be readily distinguished from the other two species in the section by the following combination of characteristics: a strongly stoloniferous growth habit, with creeping stems that serve a propagative function and terminate in a leaf rosette during fruiting; narrowly elliptic to oblanceolate or spatulate leaves; and flowers solitary in leaf axils along obliquely ascending stems. The principal morphological distinctions amongst *L. yulongensis*, *L. prolifera* and *L. pumila* are summarised in Table 1 and the key for these three species.

**Table 1.** Morphological comparison of *Lysimachia yulongensis*, *L. prolifera* and *L. pumila*.

Characters	*L. yulongensis*	*L. prolifera*	*L. pumila*
Stolons	present in the fruiting time	absent	absent
Stems	10–20 cm; the stems are mostly creeping along the ground and rooting at the nodes on the apex of the stems during fruiting time	10–28 cm, ascending to erect, often prostrate at base, lacking roots at the nodes on the apex of the stems during fruiting time	3–20 cm, decumbent or ascending, lacking roots at the nodes on the apex of the stems during fruiting time
Leaf blade	6–21 × 3–6 mm, narrowly elliptic to oblanceolate or spatulate	7–12 (–20) × 6–12 (16) mm, ovate to oblanceolate or spatulate	5–10 (–20) × 3–7 mm, spatulate to obovate or broadly ovate
Flowers	solitary in the axils of leaves, borne ascendingly along the stems	solitary in the axils of upper leaves.	capitate towards the apex
Pedicels	5–25 mm	10–15 mm	1–3 mm
Calyx lobes	3 mm	5 mm	3 mm
Corolla	white or pale pink	pink or white	pink
stamens	included, slightly shorter than corolla lobes	slightly shorter than corolla	exserted corolla
Flowering time	June–August	May-Jun	May-Jun

Most species of *Lysimachia* subgenus *Palladia* typically bear terminal racemes, with flowers seldom solitary in the upper leaf axils or clustered at the stem apex and they sustain their populations mainly through sexual reproduction. In contrast, *L. yulongensis* reproduces both sexually by seeds and clonally via stolons. Its discovery not only enhances the species diversity of the genus *Lysimachia*, but also offers new perspectives on the reproductive strategies in *L.* subgenus *Palladia*.

### Key to the species of *Lysimachia* sect. *Pumilae*

To facilitate the identification of these three species, a key is constructed as follows:

**Table:** 

1	Plants stoloniferous, rooting at nodes during fruiting; flowers solitary in leaf axils	***L. yulongensis* sp. nov**.
–	Plants prostrate or ascending, not rooting at nodes during fruiting; flowers clustered at stem apex or solitary in apical leaf axils	**2**
2	Flowers in axils of apical leaves; corolla shorter than pedicel	***L. prolifera***
–	Flowers in a terminal, subcapitate cluster; corolla longer than pedicel	***L. pumila***

## Supplementary Material

XML Treatment for Lysimachia yulongensis

## Additional information

### Use of AI

No use of AI was reported.

### Author ORCIDs

Ming-yun Sheng https://orcid.org/0009-0001-0310-4003

Wu-hai Yang https://orcid.org/0009-0007-6927-8309

Tian Shuai https://orcid.org/0009-0009-7314-8026

Yu-fan Chen https://orcid.org/0009-0001-8086-4444

Li-juan Yin https://orcid.org/0009-0004-1153-6617

Zhi-kun Wu https://orcid.org/0000-0002-4583-7782

### Data availability

All of the data that support the findings of this study are available in the main text.
